# Chimeric Antigen Receptor Expressing Natural Killer Cells for the Immunotherapy of Cancer

**DOI:** 10.3389/fimmu.2018.00283

**Published:** 2018-02-15

**Authors:** Rohtesh S. Mehta, Katayoun Rezvani

**Affiliations:** ^1^MD Anderson Cancer Center, Houston, TX, United States

**Keywords:** natural killer cells, chimeric antigen receptor, chimeric antigen receptor, chimeric antigen receptor T, chimeric antigen receptor natural killer, cancer, hematopoietic stem cell transplant

## Abstract

Adoptive cell therapy has emerged as a powerful treatment for advanced cancers resistant to conventional agents. Most notable are the remarkable responses seen in patients receiving autologous CD19-redirected chimeric antigen receptor (CAR) T cells for the treatment of B lymphoid malignancies; however, the generation of autologous products for each patient is logistically cumbersome and has restricted widespread clinical use. A banked allogeneic product has the potential to overcome these limitations, yet allogeneic T-cells (even if human leukocyte antigen-matched) carry a major risk of graft-versus-host disease (GVHD). Natural killer (NK) cells are bone marrow-derived innate lymphocytes that can eliminate tumors directly, with their activity governed by the integration of signals from activating and inhibitory receptors and from cytokines including IL-15, IL-12, and IL-18. NK cells do not cause GVHD or other alloimmune or autoimmune toxicities and thus, can provide a potential source of allogeneic “off-the-shelf” cellular therapy, mediating major anti-tumor effects without inducing potentially lethal alloreactivity such as GVHD. Given the multiple unique advantages of NK cells, researchers are now exploring the use of CAR-engineered NK cells for the treatment of various hematological and non-hematological malignancies. Herein, we review preclinical data on the development of CAR-NK cells, advantages, disadvantages, and current obstacles to their clinical use.

## Introduction

Chimeric antigen receptor (CAR) T cells have gained enormous clinical recognition with remarkable responses reported in patients receiving autologous CD19 (a B cell-specific antigen)-redirected T cells for the treatment of patients with relapsed or refractory B-cell malignancies (1–9). CAR T cells are genetically engineered to express a single chain variable fragment (scFv) derived from an antibody on their surface, which is coupled to a T-cell signaling domain, thus rendering them highly antigen-specific in a non-human leukocyte antigen (HLA)-restricted manner (2, 10, 11). Thus far, the clinical application of CAR T cells has been largely restricted to CD19-expressing B cell malignancies (1–9); however, ongoing studies are testing its applications in other hematological malignancies such as Hodgkin and non-Hodgkin lymphoma, multiple myeloma, and acute myeloid leukemia (12–14). The US Food and Drug Administration recently approved two autologous CD19 CAR T cell products for the treatment of acute lymphoblastic leukemia and certain types of relapsed or refractory large B-cell lymphoma. However, CAR T-cells have several limitations: (i) it is logistically cumbersome to generate an autologous product from patients; (ii) it takes several weeks before CAR T cells are generated—making it impractical for patients with aggressive disease; and (iii) generation of clinically relevant doses of CAR T-cells can be unfeasible from heavily pretreated lymphopenic patients. An alternative approach is to use previously collected T cells from an allogeneic source; however, even if HLA-matched, T cells pose a risk of serious graft-versus-host disease (GVHD) (15). In contrast to the popularity of CAR T cells, the generation and clinical application of CAR natural killer (NK) cells has lagged behind for various reasons, despite the multiple advantages of NK cells. Herein, we describe these barriers and discuss strategies to overcome them.

### Advantages of NK Cells for CAR Therapy

**Figure 1:**
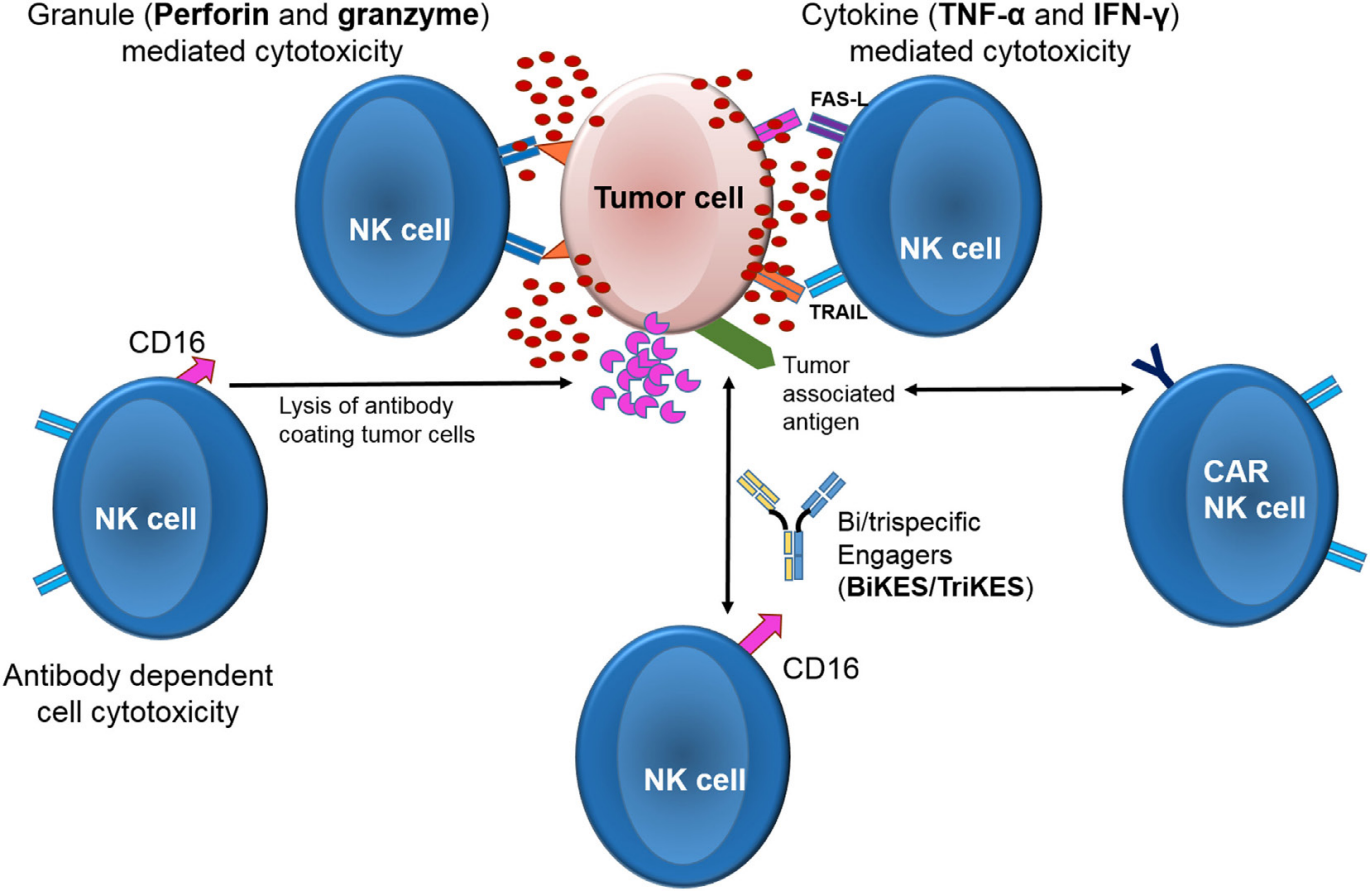
Mechanisms of action of natural killer cell cytotoxicity.

Among cytolytic lymphocytes, NK cells represent on a per cell basis the most efficient effectors against tumors with a distinct mechanism of action (Figure 1) and provide an attractive source of cells for cancer immunotherapy (16, 17). In contrast to other lymphocytes such as T or B cells, NK cells do not express rearranged, antigen-specific receptors. Instead, NK cells express germline-encoded receptors, which are either activating or inhibitory. Upon interaction with their ligands on target cells, the receptors induce a positive or a negative signal, respectively (18). The balance of these signals ultimately govern NK effector function (16, 19) Among the most heavily studied NK cells receptors are the killer-cell immunoglobulin-like receptors (KIRs) are that recognize classical HLA class-I molecules (HLA- A, -B, and -C). Other receptors belong to the C-type lectin family (CD94 and NKG2s, such as NKG2A, -B, -C, -D, -E, and -F) that recognize non-classical HLA class-I molecules (HLA-E and stress-induced MHC-I-related chains—MICA and MICB) [reviewed in Ref. (20–23)]. Healthy cells are protected from NK mediated lysis by the recognition of “self” HLA molecules on their surface by inhibitory NK receptors protects (24–27). On the other hand, tumor or viral infected cells often downregulate or lose their HLA molecules as an escape mechanism against T-cells (28, 29). Loss of HLA class I expression makes them susceptible to lysis by the NK cells due to loss of the inhibitory signal (21, 30–45). Indeed, the clinical significance of NK cell alloreactivity has been demonstrated in multiple studies in the setting of hematopoietic stem cell transplant (HSCT), where patients that received a graft containing alloreactive NK cells had a significantly lower risk of relapse and improved survival (46–55). Adoptive transfer of alloreactive NK cells as a stand-alone therapy (independent of HSCT) also demonstrated encouraging outcomes in a variety of malignancies (56–62). In contrast, several studies of *autologous* NK cell adoptive therapy showed rather disappointing results (63–71).

Thus, NK cells offer an attractive alternative to T-cells for CAR engineering for a number of reasons: (i) allogeneic NK cells should not cause GVHD, as predicted by observations in murine models (72, 73), as well as clinical studies of haploidentical and cord blood (CB)-derived NK cell infusions in patients with hematologic or solid malignancies (56, 59); (ii) mature NK cells have a relatively limited life-span, permitting effective antitumor activity while reducing the probability of long-term adverse events, such as prolonged cytopenias due to on-target/off-tumor toxicity to normal tissues such as B cell aplasia (in the case of CD19 CARs), which can last up to 3 years (74); and (iii) CAR-NK cells retain their intrinsic capacity to recognize and target tumor cells through their native receptors; therefore when compared with the CAR T cells, it is theoretically less likely for tumor cells to escape NK immunosurveillance even if they downregulate the CAR target antigen (75). This unique property of NK cells could be further exploited for the generation of NK-CARs by selecting donors based on the donor-recipient KIR-ligand mismatch, or based on donor haplotype B *KIR* gene content, as both have been shown to be beneficial in the setting of allogeneic HSCT (48, 50, 55, 76). Thus, allogeneic NK cells offer the potential for an off-the-shelf cellular product for immunotherapy that could be readily available for immediate clinical use, in contrast to the current shortage of CAR T-cell products at many centers (77).

## Source of NK Cells for Adoptive Immunotherapy

Functional NK cells can be generated from numerous sources. Although autologous NK cells can be utilized for adoptive therapy, their efficacy against autologous cancer cells is rather limited (63–71, 78, 79), which we have shown may not be easily overcome by CAR engineering (80). Allogeneic NK cell sources include peripheral blood (PB), bone marrow (BM), human embryonic stem cells (hESCs), induced pluripotent stem cells (iPSCs) (81–83), umbilical CB, or readily available NK cell lines (84). Obtaining NK cells from the PB by apheresis or from BM by harvesting are both cumbersome and are associated with potential risks to the healthy donors (85–87). NK cell derivation from hESCs or iPSCs (81–83) is a complex process and the field is still evolving. In contrast, NK cell lines such as NK-92 (88–93), KHYG-1 (94), NKL, NKG, and YT, to name a few, provide an easily accessible and homogeneous source of cells for the generation of large numbers of CAR-transduced NK cells. NK-92 is a highly cytotoxic NK cell line that was derived from a patient with NK lymphoma (95) and is characterized as CD56^bright^CD16^neg/low^NKG2A^positive^ and KIR^negative^ (except for KIR2DL4) (96, 97). Phase I clinical studies demonstrated the safety of NK-92 cell infusion in cancer patients, even up to doses of 10^10^ cells/m^2^ (98–100). Based on these data, there is great interest in CAR-engineered NK-92 cells for clinical use (Table 1) (88–92, 101–115). However, NK-92 cells have a number of disadvantages that need to be taken into account. First and foremost, NK-92 cells are derived from a patient with NK lymphoma (95) and thus have the potential for tumor engraftment following infusion. Moreover, they are EBV-positive and carry multiple cytogenetic abnormalities resembling those of NK lymphoma (116). Thus, as a safety measure, NK-92 cells must be irradiated before infusion into patients to prevent permanent engraftment. This can negatively impact their *in vivo* proliferation and persistence, both factors shown to be crucial for the success of cellular therapy in studies with infusion of tumor-infiltrating lymphocytes (117–119) as well as CAR-T cells (3). Indeed, in a study of NK-92 cells engineered with ErbB2/HER2-CAR, while irradiation had no effect on the *in vitro* cytotoxicity of CAR-transduced NK92 cells, it negatively impacted their *in vivo* replication and persistence, with the cells no longer detectable within 7 days of adoptive infusion (109). Of note, NK-92 cells are CD16 (FCRIIIγ) negative and cannot mediate antibody-dependent cell cytotoxicity (ADCC), unless genetically modified to express CD16 (120).

**Table 1:** Clinical trials with NK CAR.

Clinical trial identifier	NK cell source	Target antigen	Disease	Study location
NCT02944162	NK-92 cell line	CD33	AML	China
NCT02892695	NK-92 cell line	CD19	CD19 positive B cell malignancies	China
NCT02742727	NK-92 cell line	CD7	CD7 positive leukemia or lymphoma	China
NCT02839954	NK-92 cell line	MUC1	MUC1 positive solid tumors (colorectal, gastric, pancreatic, NSCLC, breast, glioma)	China
NCT03056339	Cord blood	CD19	CD19 positive leukemia or lymphoma	MDACC, USA

*AML, acute myeloid leukemia; MDACC, MD Anderson Cancer Center; NK, natural killer; NSCLC, non-small cell lung cancer*.

Cord blood, on the other hand, is a readily available source of allogeneic NK cells with distinct benefits over related or unrelated adult donors, including the speed of availability (especially since it is available as an off-the-shelf frozen product) and tolerance of HLA mismatches, the latter of which expands the donor pool. The frequencies of NK cells in CB (~15–20%) are similar to PB (~10–15%) (121–124). However, until recently the small volume of blood in a CB unit made it challenging to obtain adequate numbers of NK cells for clinical use. Moreover, resting CB NK cells are phenotypically and functionally immature, with higher expression of the inhibitory receptor NKG2A and lower expression of activating and maturation receptors such as NKp46, NKG2C, DNAM-1 (124), and CD57 (124–127). To overcome these limitations, our group has developed a Good Manufacturing Practice (GMP)-compliant procedure, using GMP-grade K562-based artificial antigen-presenting cells (aAPCs) expressing membrane bound IL-21 and 4-1BB ligand, which reliably generates clinically relevant doses of GMP-grade NK cells from a CB unit for adoptive immunotherapy (128). Following *ex vivo* activation and expansion, CB-derived NK cells display the full array of activating and inhibitory receptors, strongly express eomesodermin (Eomes) and T-bet, two factors necessary for NK cell maturation, and exert similar cytotoxicity to PB-NK cells (129, 130). Taken together, these studies support the use of NK cells as a source of cellular therapy in cancer.

### Constituents of CAR

A CAR construct consists of three components: an extracellular antigen-recognition part, a transmembrane domain and an intracellular signaling domain. The extracellular domain is the antigen-recognition site and is generally composed of an scFv derived from the variable regions of both the heavy and light chains of a monoclonal antibody, fused together *via* a flexible linker. Most scFvs studied to date are of murine origin, with the potential to induce a human antimouse antibody (HAMA) or an anti-idiotype immune response. A number of investigators are exploring strategies to humanize scFVs (131–136) to circumvent induction of HAMA; however, this approach will not prevent the development of anti-idiotype antibodies. The antigen binding domain of a CAR is linked to a “hinge” which imparts flexibility for adequate orientation and binding to the antigen. The hinge binds the extracellular component to a transmembrane domain, which is the link to the intracellular signaling component (137–139). The size of the hinge region has been shown to affect CAR-T cell function, with some studies reporting superior anti-tumor activity of CAR T-cells expressing a shorter hinge (140, 141). The transmembrane domain lies between the hinge and the signaling endodomains. Different types of transmembrane domains have been studied, including the CD3-ζ chain of the T-cell receptor, CD4, CD8, or CD28. The type of transmembrane domain has also been shown to affect the function and stability of the CAR molecule in T cells (142). The endodomain then transmits activation signals to T cells. The “first-generation” CARs used a single intracellular signaling domain (CD3-ζ chain alone) while the second- and third-generation CARs incorporate one or more additional costimulatory signaling domains, such as CD28, CD137, or OX40 to render them more potent (143, 144).

CD3ζ is critical for signaling and activation of both T and NK cells (145). In NK cells, CD3ζ homodimer transmits signals from FcγRIII (CD16), thus aiding in ADCC (146). Although CD28 is one of the most commonly employed costimulatory domains in CAR T cells, except in certain cell lines (147), its role in NK cell function is less clearly defined (148). Nonetheless, its addition to CD3ζ in a second generation ErbB2-specific NK-92 CAR led to improved function compared to a CD3ζ construct alone and was similar to that of CD137-CD3ζ CAR against ErbB2-expressing tumor cells (109). Another study showed that NK-92 cells transduced with a CD19-CAR expressing CD28-CD3ζ had superior cytotoxicity against CD19-positive targets compared to cells expressing a CD137-CD3ζ containing CAR (88). DNAX-activation protein 12 (DAP12) is transmembrane protein involved in signal transduction of several NK cell activating receptors including NKG2C, NKp44, and the activating KIRs (149). One study tested if a CAR against prostate stem cell antigen (PSCA) that used DAP12 as an intracellular signaling domain can provide sufficient signaling to induce NK cell activation when compared to a CD3ζ-containing CAR. The authors transduced YTS-NK cells and primary NK cells with PSCA-DAP12 CAR and noted superior cytotoxicity when compared to NK cells expressing a CD3ζ-based CAR (150). While the importance of incorporating costimulatory molecules in the CAR construct has been clearly shown for CAR T cells (4, 5, 151), additional studies are needed to define the optimal costimulatory molecule and signaling endodomain for NK cells.

### CAR Transduction

The incorporation of a foreign gene into a cell requires the use of a vector, which can be based on viral or non-viral systems. The most commonly used tools for CAR gene delivery include genetically engineered retroviruses [lentiviral (152) and gamma-retroviral (153) vectors]. Lentiviruses have the advantage that they are capable of infecting both dividing and non-dividing cells, while retroviruses only infect dividing cells. Therefore, lentiviral vectors can be used for transduction of a wider variety of cell types including quiescent stem cells (154–156). In addition, lentiviral vectors can accommodate larger transgenes when compared with retroviral vectors (157). Insertional mutagenesis, although extremely rare, remains a concern with viral vectors although its likelihood is influenced by a number of factors such as the specific type of vector used and the site of integration (158). For instance, in earlier trials of gene therapy with CD34^+^ hematopoietic cells for X-linked severe combined immunodeficiency (SCID), 2 of the 10 treated children developed acute leukemia in one study (159) and 1 of the 4 children in another study (160). However, none of the subsequent trials of gene-modified hematopoietic stem cells in SCID children (161) or studies of adoptive immunotherapy with CAR T-cells (2, 3, 5, 9, 162) have witnessed adverse events related to insertional mutagenesis to date. Yet, to mitigate any theoretical concerns, various non-viral techniques such as the transposon/transposase system (82, 163) or mRNA transfection (113, 115) have also been tested. The transduction efficiency using these techniques varies remarkably from study to study (82, 113, 115, 163, 164) and depends on a number of factors, including the cell source (82, 113, 115, 163–167). In general, the transduction efficiency for CAR T cells is about 50% but can range up to 90% or higher (152, 164, 168).

## Challenges

Despite the many advantages of NK cells, there are several impediments to the successful generation of CAR NK cells for clinical use. Until recently, the genetic engineering of NK cells, even with viral methods, had proved challenging, with reports of <10% transduction efficiency for primary CB or PB derived NK cells (113, 165). However, recent optimization in protocols for viral transduction and electroporation (166, 167) has revived enthusiasm for the genetic engineering of NK cells. While viral methods appear to be largely ineffective for inducing CAR expression in freshly isolated PB NK cells, significantly better transduction efficiency can be achieved when NK cells from PB (12–73%) (113) or CB are activated and expanded (median 69%; range 43–93%) (169) in one study and 80% (range 67–96%) (170) in another study. In contrast to studies with primary NK cells, NK92 cells are easier to transduce with mRNA electroporation (113, 115), with efficiencies averaging from 25 to 50% (171). However, as the mRNA transcript is not incorporated into the genome, expression of the CAR molecule is often short-lived and detectable for only a few days, which may negatively impact the efficacy of the engineered cells following adoptive transfer (115, 166, 167).

Another concern with using allogeneic NK cells is the possibility of infusing contaminating T or B cells in the expanded NK cell product, which can theoretically cause GVHD or posttransplant lymphoproliferative disease, respectively. As some degree of HLA-mismatch after CB transplantation is well tolerated, the risk of clinically significant GVHD may be less with CB-derived CAR-NK cells compared to PB. Plus, with the exception of one study reporting GVHD following adoptive transfer of donor-derived IL-15/4-1BBL-activated NK cells in recipients of HLA-matched, T-cell-depleted PB HSCT (72), clinical studies of haploidentical and CB NK cell infusions in hundreds of patients with both hematologic and solid malignancies have not reported a higher risk of GVHD (56, 58, 128, 172–174). Rather, experimental evidence obtained in mice have reported reduced risk of GVHD with NK cells *via* multiple mechanisms, including depletion of host antigen-presenting cells and activated alloreactive T cells (46, 72, 175, 176). Another potential limitation of NK cells for immunotherapy is that in contrast to T cells, they are highly sensitive to the freeze and thaw process and they lose activity after thawing. A number of groups are exploring strategies to optimally cryopreserve NK cells and have shown that the activity of frozen NK cells can be restored by overnight incubation with cytokines such as IL-2 (177–182). It is not yet known if a similar strategy can be used to restore function of frozen CAR NK cells for adoptive therapy.

Another characteristic of NK is that they do not persist after adoptive transfer without cytokine support (183). While the shorter life-span of NK cells may be advantageous, allowing for antitumor activity while reducing the probability of long-term adverse events such as prolonged cytopenias caused by on-target/off-tumor toxicity to normal tissues, it may also limit their efficacy. For *in vivo* survival and proliferation, NK cells require continuous cytokine support, without which they are detectable in the circulation for only 1–2 weeks (183). The two most commonly used cytokines to support the persistence of adoptively transferred NK cells are IL-2 and IL-15 (184, 185). The infusion of IL-2 has substantial side effects including fevers, chills, myalgias and capillary leak syndrome (186), and can promote expansion of regulatory T cells (T_regs_) which are suppressive to NK cells (187). IL-15, on the other hand, does not support T_reg_ (188) expansion but when administered as an exogenous bolus to patients with metastatic melanoma and renal carcinoma can result in dose-dependent toxicity, including neutropenia (189). An alternative approach to exogenous administration of cytokines is to treat patients with lymphodepleting chemotherapy such as cyclophosphamide and fludarabine prior to infusion of NK cells, which provides a favorable environment for NK cell expansion by depleting mature lymphocytes (which consume IL-15), resulting in a marked increase in endogenous IL-15 levels (56). Another novel technique is to incorporate genes for IL-2 (104, 190–192) or IL-15 (80, 193–195) within the CAR construct to constantly provide cytokine support to the CAR-transduced cells. We recently showed the feasibility and efficacy of this approach in a mouse model of Raji lymphoma. Although a single infusion of 1 × 10^7^ CAR.19^+^ (without IL15) or CAR.19/IL15^+^ CB-NK cells both improved tumor control and prolonged survival compared to non-transduced CB-NK, CAR.19/IL15^+^ CB-NK cells controlled tumor expansion and prolonged survival significantly better than the CAR.CD19 construct lacking the *IL-15* gene, which underscores the critical influence of IL-15 in enhancing antitumor activity *in vivo* (80).

## Suicide Genes

Given the recent safety concerns such as cytokine release syndrome and neurotoxicity associated with infusion of CAR-modified T cells (196, 197) (and possibly NK cells), careful consideration of whether a suicide system should be incorporated into the construct as a safety measure is needed. One of the most extensively tested safety switches include the herpes simplex virus thymidine kinase gene (198, 199). While a number of studies have tested this approach, the highly immunogenic virus-derived protein can lead to the rejection of cells expressing it, plus it requires administration of ganciclovir—which takes several days to work and leads to cytopenias (200–202). Because of these disadvantages, inducible caspase-9 (iCasp9) has emerged as one of the most commonly used suicide genes in adoptive cell therapy trials (80, 193, 203–206). When exposed to a synthetic bioinert small-molecule dimerizing drug, the iCasp9 becomes activated and leads to rapid apoptosis of cells expressing it. Another suicide gene under investigation is the truncated epidermal growth factor receptor (EGFR), which lacks intracellular tyrosine kinase activity while expressing an intact binding epitope that can be targeted with the anti-EGFR monoclonal antibody cetuximab for the rapid elimination of the transgenic cells (207, 208).

### Preclinical Studies of CAR-NK Cells

Building on the knowledge gained with CAR T-cells, a multitude of preclinical studies have tested the efficacy of CAR NK cells against a variety of target antigens for hematological malignancies such as CD19 (167, 169), CD20 (209, 210), CD138 (211), CS1 (111), CD3 (212), CD5 (101), CD123 (213), as well as solid tumors such as HER-2/Erb-2 (109, 214–216), GD2 (114), EpCAM (195), EGFR and mutant EGFRvIII (89), WT1 (217), and ROR-1 (218) to name a few. An alternative and non-antigen specific approach to engineering NK cells was tested by Chang et al. (170), where the authors induced supra-physiologic expression of NKG2D, a key receptor for NK cell activation and signal transduction *via* DNAX-activating protein 10 (DAP10). *Ex vivo* expanded PB NK cells transduced with a retroviral vector encoding NKG2D-DAP10-CD3ζ showed impressive *in vivo* cytotoxicity in xenogeneic mouse models of hematologic and solid tumors but showed no activity against non-transformed blood or mesenchymal cells (170).

Despite these impressive preclinical data, there are currently only five registered clinical trials testing the safety and efficacy of CAR-NK cells in cancer patients (Table 1). Four of these trials are being conducted in China using the CAR-engineered NK92 cells. The only trial using primary NK cells (CB NK cells) is being conducted in the United States by our group at the MD Anderson Cancer Center (NCT03056339). Patients with relapsed or refractory CD19^+^ B cell lymphoid malignancies are eligible for this trial. All patients receive lymphodepleting chemotherapy with fludarabine and cyclophosphamide, followed by the infusion of allogeneic CB-derived NK cells that are genetically modified with a retroviral vector, iC9-2A-CAR.CD19-CD28-CD3zeta-2A-OhIL-15 (iC9/CAR.19/IL15) (80), which (i) includes CAR.19 gene to redirect specificity to CD19; (ii) produces IL-15 ectopically—a cytokine crucial for NK cell survival and proliferation (219); and (iii) incorporates inducible caspase-9 (*iCasp9*)—a suicide gene, which can be activated pharmacologically to eliminate CAR cells, as needed (203, 219).

## Conclusion

We are in an exciting era in the field of cellular therapy. NK cells hold great promise and offer the potential for an off-the-shelf cellular product for immunotherapy that could be readily available for immediate clinical use. Yet, before NK cells can be extended to larger cohorts of patients a number of scientific questions and regulatory hurdles must be addressed. What is the ideal vector, signaling endodomain and costimulatory molecule for NK cells—one with the best response and safety profile? Will CAR NK cells have a different safety and efficacy profile to CAR T cells, given their distinct mechanism of action? Will “off-the-shelf” CAR NK cells be able to sustain the clinical demand, give the shortage of CAR-T cells at many centers and the uncertainty regarding the health economics of this treatment? Can CAR NK cells lead to durable responses, considering their limited *in vivo* life-span or will they be used as a “bridge” to more aggressive treatment such as HSCT? Based on the results of haploidentical and unrelated donor HSCT, should NK cells for CAR modification be selected based on KIR-KIR ligand mismatch or KIR haplotype to harness their native NK cell activity and will this approach reduce the risk of disease escape through downregulation of the CAR target antigen? With the plethora of preclinical studies and clinical research that are underway, it is expected that engineered NK cells will make a significant contribution to the recent paradigm shift in cancer treatment.

## Author Contributions

RM and KR reviewed the literature, analyzed data, and wrote the manuscript.

## Conflict of Interest Statement

The authors declare that the research was conducted in the absence of any commercial or financial relationships that could be construed as a potential conflict of interest.
